# Maternal near miss determinants at a maternity hospital for high-risk pregnancy in northeastern Brazil: a prospective study

**DOI:** 10.1186/s12884-019-2381-9

**Published:** 2019-08-01

**Authors:** Telmo Henrique Barbosa de Lima, Melania Maria Amorim, Samir Buainain Kassar, Leila Katz

**Affiliations:** 1 Health Sciences University of Alagoas (UNCISAL), Rua Dr. Mario Nunes Vieira, 149 – Apto. 201, Jatiuca, Maceió, AL Brazil; 2 Prof. Fernando Figueira Institute of Integral Medicine (IMIP), Department of Obstetrics and Gynecology, Recife, Brazil; 3 Health Sciences University of Alagoas (UNCISAL), Maceió, Brazil; 4 Prof. Fernando Figueira Institute of Integral Medicine (IMIP), Obstetric Intensive Care Unit, IMIP, Recife, Brazil

**Keywords:** Maternal near miss, Maternal mortality, Severe maternal morbidity

## Abstract

**Background:**

To investigate the association between sociodemographic and obstetric variables and delays in care with maternal near misses (MNMs) and their health indicators.

**Methods:**

A prospective cohort study was conducted at a high-risk maternity hospital in northeastern Brazil from June 2015 to May 2016 that included all pregnant women seen at the maternity hospital during the data collection period and excluded those who had not been discharged at the end of the study or whom we were unable to contact after the 42nd postpartum day for MNM control. We used the MNM criteria recommended by the WHO. Risk ratios (RRs) and their 95% confidence intervals (CIs) were calculated. Hierarchical multiple logistic regression analysis was performed. The *p* values of all tests were two-tailed, and the significance level was set to 5%.

**Results:**

A total of 1094 pregnant women were studied. We identified 682 (62.4%) women without adverse maternal outcomes (WOAMOs) and 412 (37.6%) with adverse maternal outcomes (WAMOs), of whom 352 had potentially life-threatening conditions (PLTCs) (85.4%), including 55 MNM cases (13.3%) and five maternal deaths (1.2%). During the study period, 1002 live births (LBs) were recorded at the maternity hospital, resulting in an MNM ratio of 54.8/1000 LB. The MNM distribution by clinical condition identified hypertension in pregnancy (67.2%), hemorrhage (42.2%) and sepsis (12.7%). In the multivariate analysis, the factors significantly associated with an increased risk of MNM were fewer than six prenatal visits (OR: 3.13; 95% CI: 1.74–5.64) and cesarean section in the current pregnancy (OR: 2.91; 95% CI: 1.45–5.82).

**Conclusions:**

The factors significantly associated with the occurrence of MNM were fewer than six prenatal visits and cesarean section in the current pregnancy. These findings highlight the need for improved quality, an increased number of prenatal visits and the identification of innovative and viable models of labor and delivery care that value normal delivery and decrease the percentage of unnecessary cesarean sections.

## Background

From 1990 to 2015, maternal mortality dropped approximately 44% worldwide, which, although positive, was insufficient to reach the Millennium Development Goals. From 2016 to 2030, the goal of the United Nations 2030 Agenda for Sustainable Development is to reduce the global maternal mortality rate to fewer than 70 per 100,000 live births [1, 2].

Although catastrophic, maternal death is numerically rare in high-income regions. In developing countries, though more common it is scattered and sporadic in numerous health institutions, which together with underreporting complicates and increases the costs of epidemiological studies to determine risk factors and determinants. Studying women who survive severe complications during the pregnancy and postpartum has been a way suggested to overcome this challenge [3].

Women who survive severe pregnancy complications have attracted the interest of researchers and public policymakers since the 1990s. This group, which is known as maternal near misses (MNMs), is formed by women who escape death after an acute and severe pregnancy complication [4].

In 2009, the WHO established a technical working group consisting of obstetricians, midwives, epidemiologists and public health professionals to develop a standard definition of and uniform identification criteria for MNMs. This concept has since been attributed to cases of women who survive a severe, life-threatening complication during pregnancy, childbirth or within 42 days after delivery who meet any of the clinical, laboratorial or management criteria advocated by the WHO [5].

The WHO recommends that the MNM approach be considered in national plans to improve maternal health, because researchers agree that MNMs are frequently a preventable precursor of maternal mortality [6]. Using the same classification, countries and regions of the same country can be compared to help identify deficiencies in the healthcare system, improve the quality of care during the pregnancy-puerperal cycle and guide more recent studies on the topic [5].

Another strategy for reducing severe maternal outcomes is identification of possible failures in the care provided to these women by the healthcare system. Studies have shown that many pregnant women arrive at health services in such precarious conditions that they cannot be saved and that the time needed to receive adequate care is the most important factor in their deaths [7].

Assuming that most of these outcomes are preventable, they may be explained by the “three delays model”. This model aims to identify when maternal care fails to create death prevention strategies. The first delay occurs when the patient or relatives are unable to seek care, the second when the patient struggles to access a health facility after deciding to seek care and the third when healthcare professionals are unable to recognize or initiate the care required in time or when the health facility lacks adequate infrastructure for care [7].

We conducted an active Internet search in the MEDLINE, BIREME and SCOPUS databases using the descriptor “maternal near miss”. Despite the increasing number of studies on the topic, no studies have addressed MNMs in the region of Alagoas, Brazil. Therefore, the aim of the present study was to identify the MNM determinants in the Alagoas region.

## Methods

### Study setting

A prospective cohort observational analytical study was conducted at the Santa Mônica University Maternity Hospital (Maternidade Escola Santa Mônica – MESM) located in the municipality of Maceió, which is the capital of the state of Alagoas, Brazil. This center is a high-risk maternity hospital that exclusively treats pregnant women through the Unified Health System (Brazil’s public health system). This hospital is the main tertiary referral center for high-complexity obstetric and neonatal care in Alagoas.

Data were collected from June 2015 to May 2016 by the principal investigator and research assistants from the Medicine Program of Alagoas State University of Health Sciences (Universidade de Ciências da Saúde de Alagoas – UNCISAL). The study was conducted after approval by the UNCISAL Human Research Ethics Committee under CAAE n. 37,977,014.0.0000.5011. All puerperal women included in the study voluntarily agreed to participate and signed the informed consent form.

### Study population and selection

The study population consisted of 1149 pregnant women seeking treatment at the hospital during the data collection period. In total, 1094 women admitted to the hospital, and that had not been discharged by the end of the study or whom we were unable to contact after the 42nd postpartum day for MNM control, were eligible for the study. The patients that died or that had potentially life-threatening conditions were excluded.

TheMNM selection criteria were defined according to the criteria advocated by the WHO [5]. The women who met the inclusion criteria were interviewed using pretested questionnaires, and relevant data were also collected from their medical records. A second interview was conducted after the 42nd postpartum day to assess the maternal outcome.

### Statistical analysis

The statistical analysis was performed using the Epi Info 3.5.1 (Atlanta, GA). Some WHO indicators of maternal care quality [5] were used, including the potentially life-threatening conditions ratio (PLTCR), maternal near miss ratio (MNMR), maternal death ratio (MDR), severe maternal outcome ratio (SMOR), maternal near miss ratio, mortality index (MI) and live births (LBs).

In the bivariate analysis, MNM was the outcome variable, and all other variables were considered exposure variables and categorized as dichotomous (yes or no) variables. The risk ratio (RR) was calculated as a risk measure with a 95% confidence interval (95% CI). The Chi-square or Fisher’s exact test and the Mann-Whitney U test were used when appropriate.

All variables analyzed in the bivariate analysis were included in the multiple logistic regression analysis to identify the variables most strongly associated with MNMs after determining the adjusted risk. A hierarchical model was constructed for the multiple regression analysis, and the variables were positioned in blocks; the most distal factors were the biological and socioeconomic variables non-Caucasian ethnicity, education <8 years, countryside origin, family income of 1 minimum wage or less, age < 19 years, age > 35 years and single marital status. Variables related to prenatal care and delivery were included in the intermediate level, including no prenatal care, <6 prenatal visits, no prenatal care at the maternity clinic, no delivery referral and cesarean section in a previous pregnancy. The variables considered closest to the MNM outcome were included in the proximal level, such as presence of comorbidity, cesarean section in the current pregnancy, some delay in care and 1st, 2nd and 3rd delays in care.

Stepwise logistic regression was performed. The variables significantly associated with the outcome at the end of each block at a 20% level and then those that remained associated with the outcome at a 5% significance level were highlighted. A final regression analysis was performed to determine the adjusted risk of MNMs for each variable significantly associated with the outcome at the 5% significance level.

## Results

**Fig. 1:**
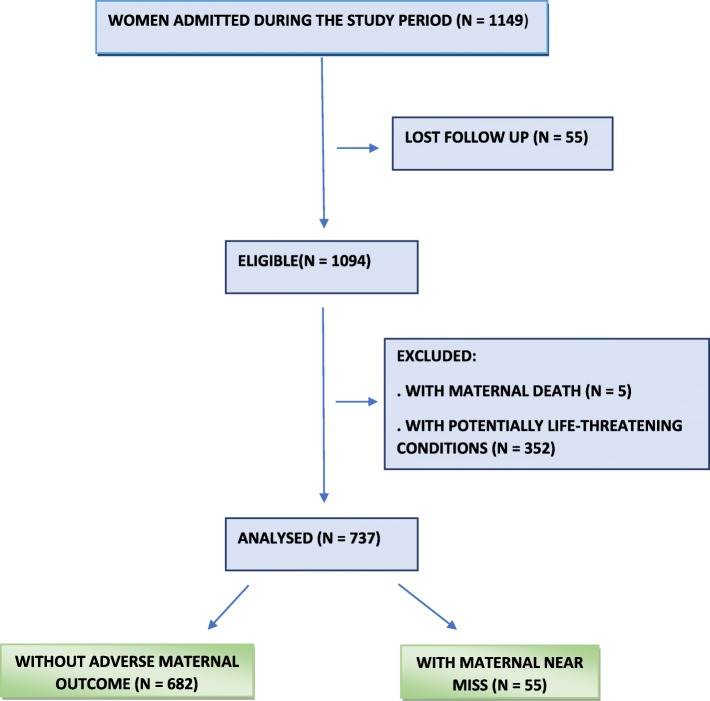
Flow-chart of participants

From June 2015 to May 2016, 1149 women were admitted to the unit. During follow up, 55 were lost, so 1094 pregnant women were eligible. Five maternal deaths occurred, and 352 women had potentially life-threatening conditions and were also excluded from the final sample. For analysis 737 patients were included, 682 women without adverse maternal outcome (WOAMOs) were compared to 55 women with maternal near miss (MNM) (Fig. 1). During the study period, 1002 live births occurred in the maternity hospital, reaching an MNM ratio of 54.8/1000 LBs and an MNM/maternal death ratio of 11/1000 LB (Table 1).

**Table 1:** Maternal near miss indicators

Indicators	N	Rate
Number of live births	1002	
Potentially life-threatening conditions (PLTCs)	352	
Women with maternal near misses	55	
Maternal death	5	
Maternal near miss ratio		54.8/1000 LBs
Severe maternal outcome ratio		57/1000 LBs
Maternal death rate		99/100,000 LBs
MNM/Maternal Death Ratio		11/1000 LBs
Mortality Index		8.33%

*MNM* maternal near miss, *LB* live birth

Clinical criteria defined by WHO were identified in 50 patients (90.9%). A respiratory rate > 40 or < 6 L/min, which was identified in 18 patients (40.0%), was the most frequent clinical criterion, with aMNM ratio of 21.9/1000 LBs. Management criteria were the second most frequent and were identified in 39 patients (70.9%). Packed red blood cell transfusion was the most frequent management criterion and was identified in 29 patients (49.0%), with an MNM ratio of 26.9/1000 LBs. Laboratory criteria were identified in 18 patients (32.7%), of which a low platelet count was the most commonly diagnosed event [identified in 13 patients (23.6%) with an MNM ratio of 12.9/1000 LBs]. Of the women with MNMs, 19 met one diagnostic criterion (34.5%), 23 met two criteria (41.8%), and 13 met three criteria (23.6%) (Table 2).

**Table 2:** Incidence and distribution of maternal near miss cases according to criteria defined by the World Health Organization (WHO)

	n	%	Incidence of maternal near miss/ 1000 live births = 54.8
Number of criteria observed			
1	19	34.5	18.9
2	23	41.8	22.9
3	13	23.6	12.9
Clinical criteria	50	90.9	49.9
Acute cyanosis	18	32.7	17.9
Gasping	05	9.0	4.9
Shock	20	36.3	19.9
Respiratory rate > 40 or < 6 L/min	22	40.0	21.9
Non-responsive oliguria	01	1.8	0.9
Coagulopathy	04	7.2	3.9
Convulsions	17	30.9	16.9
Jaundice in the presence of pre-eclampsia	05	9.9	4.9
Laboratory criteria	18	32.7	17.9
Total bilirubin ≥6.0 mg/dL	01	1.8	0.9
Creatinine ≥3.5 mg/dL	01	1.8	0.9
PaO**2**/FiO**2** < 200 mmHg	01	1.8	0.9
Low platelet count (< 50,000 platelets)	13	23.6	12.9
O**2** saturation < 90% for ≥60 min	06	10.9	5.9
Management criteria	39	70.9	38.9
Dialysis for acute renal failure	01	1.8	0.9
Hysterectomy for infection or hemorrhage	09	16.3	8.9
Intubation and mechanical ventilation	17	30.9	16.9
Packed red blood cell transfusion	27	49.0	26.9
Continuous vasoactive drug support	15	27.2	14.9

The MNM distribution by clinical condition resulted in 37 cases of hypertension in pregnancy (67.2%), 26 cases of hemorrhage (42.2%) and 7 cases of sepsis (12.7%). Eclampsia was the most frequent hypertensive disorder (16.9/1000 LBs), placental abruption (PA) (7.9/1000 LBs) had the highest incidence among antepartum hemorrhage, and uterine atony (5.9/1000 LBs) was the most frequent condition among postpartum hemorrhages (Table 3).

**Table 3:** Incidence and distribution of maternal near miss cases according to clinical conditions

Clinical conditions	n	%	Maternal near miss incidence/1000 live births = 54.8
Hypertension in pregnancy	37	67.2	36.9
Severe preeclampsia	15	27.2	14.9
Eclampsia	17	30.9	16.9
HELLP syndrome	05	9.1	4.2
Hemorrhage	26	42.2	25.9
Antepartum hemorrhage	15	27.2	14.9
Placental abruption	08	14.5	7.9
Ectopic pregnancy	07	12.7	6.9
Postpartum hemorrhage	11	20.0	10.9
Atonia	06	10.9	5.9
Coagulopathy	03	5.4	2.9
Retained placenta	02	3.6	1.9
Sepsis	07	12.7	6.9
Acute pulmonary edema	01	1.8	0.9
Respiratory failure	01	1.8	0.9

*HELLP* hemolysis, elevated liver enzymes, low platelet count

The bivariate analysis between sociodemographic and obstetric variables and delays in care obtained the following significant associations: no pre-natal visits (RR: 3.77; 95% CI: 2.05–6.93; *p* = 0.00002); fewer than six prenatal visits (RR: 2.45; 95% CI: 1.43–4.19; *p* = 0.0006); no delivery referral (RR: 2.24; 95% CI: 1.14–4.37; *p* = 0.01); cesarean section in the current pregnancy (RR: 2.20; 95% CI: 1.16–4.11; *p* = 0.01); some delay in care (RR: 2.31; 95% CI: 1.33–4.02; *p* = 0.002) and 2nd delay in care (RR: 1.89; 95% CI: 1.14–3.15; *p* = 0.01). The other variables showed no significant differences between groups (Table 4).

**Table 4:** Sociodemographic and obstetric characteristics and delays in care associated with maternal near miss and pregnant women without adverse outcomes – bivariate analysis

Variables	MNM	WOAMO	RR (95% CI)	p
TOTAL	55(7.5)	682(92.5)		
Distal factors				
Age (< 20 years)	19(34.5)	208(30.5)	1.18(0.69–2.02)	0.53
Age (> 35 years)	05(9.1)	88(12.9)	0.69(0.28–1.69)	0.41
Non-Caucasian ethnicity	43(78.2)	546(80.1)	0.90(0.48–1.66)	0.73
Education < 8 years	29(52.7)	360(52.7)	0.99(0.59–1.66)	0.99
Rural origin	28(50.9)	344(50.4)	1.01(0.61–1.69)	0.94
Family income (1 or less MW)	46(83.7)	551(80.8)	1.19(0.60–2.39)	0.60
Marital status single	16(29.1)	141(20.7)	1.51(0.87–2.63)	0.14
Intermediate factors				
**Without prenatal (PN) visits**	**10(18.2)**	**31(4.5)**	**3.77(2.05–6.93)**	**0.00002**
**Fewer than 6 PN visits**	**36(65.5)**	**512(75.1)**	**2.45(1.43–4.19)**	**0.0006**
No prenatal care at the center	37(67.3)	512(75.1)	0.70(0.41–1.20)	0.20
No house calls	54(98.2)	661(96.9)	1.66(0.24–11.47)	0.50*
**No delivery referral**	**45(81.8)**	**447(65.5)**	**2.24(1.14–4.37)**	**0.013**
Previous cesarean section	29(52.7)	388(56.9)	0.85(0.51–1.42)	0.54
Proximal factors				
**Current cesarean section**	**44(80.0)**	**431(63.2)**	**2.20(1.15–4.19)**	**0.01**
Comorbidities (Yes)	21(38.2)	253(37.1)	1.04(0.61–1.76)	0.87
**Some delay**	**38(69.1)**	**324(47.6)**	**2.20(1.15–4.19)**	**0.002**
1st delay	07(12.7)	95(1.9)	0.90(0.42–1.95)	0.80
**2nd delay**	**29(52.7)**	**244(35.8)**	**1.89(1.14–3.15)**	**0.01**
3rd delay	11(20.0)	93(13.6)	1.52(0.81–2.84)	0.19

*Fisher

After the multivariate analysis, the factors significantly associated with an increased risk of MNM were fewer than six prenatal visits (OR: 3.13; 95% CI: 1.74–5.64; *p* = 0.0001) and cesarean section in the current pregnancy (OR: 2.91; 95% CI: 1.45–5.82 *p* = 0.0024) (Table 5).

**Table 5:** Multivariate analysis of MNM determinants

	Odds ratio	95% CI	*p*
Intermediate factors			
<6 prenatal visits	3.13	1.74–5.64	0.0001
Proximal factors			
Current cesarean section	2.91	1.45–5.82	0.0024

## Discussion

In the present study, the MNM ratio was 54.8/1000 LBs when the WHO criteria [5], which identify the most severe cases with the highest risk of death [8], were used. The MNM ratio varies worldwide according to the study region and hospital type. This finding is compatible with the wide spectrum of incidence of MNMs evidenced in the last systematic review [9]. A study conducted at a tertiary referral hospital in Teresina, Piauí, Brazil, reported an MNM ratio of 9.6/1000 LBs [10]. A national hospital-based study conducted in Brazil with 23,894 women reported an MNM ratio of 10.2/1000 LBs [11], and a study performed in Ethiopia in 2017 reported an estimated MNM ratio of 8.01/1000 LBs [12].

The almost five-fold higher incidence of MNMs recorded in our study might be explained by the fact that this study was conducted in a referral maternity hospital that only treated high-risk pregnant women. Consequently, the sample was selected by severity and by a higher frequency of MNMs than that expected for other health services [13].

Another indicator we used to monitor the quality of obstetric care was the MNM/maternal mortality ratio, which was 11/1000 LBs in our study, showing the occurrence of one maternal death for every 11 MNM cases. The MNM/maternal mortality ratio reported in the literature using the WHO criteria is at least four cases of MNM for every maternal death [8, 14, 15]. The higher proportion in our study might be explained by the fact that our MNM ratio was high and therefore this group could include a higher number of less severe cases and consequently higher survival. Another factor is that 94.5% of the pregnant women with MNMs were treated in our obstetric ICU by healthcare professionals trained for obstetric emergencies, which might have been an important differential for maternal survival.

This finding is an important indicator that justifies studying MNMs in audits, because their frequency is much higher than maternal mortality and thus provides a higher number of patients with similar characteristics. Furthermore, MNMs are frequently preventable precursors of maternal death [6].

The characterization of MNM cases advocated by the WHO uses the diagnosis of organ dysfunction, which may be characterized by clinical, laboratorial and management criteria. In our study, clinical criteria were the most frequent, followed by management and laboratorial criteria. In a study assessing data on the MNM incidence [the *Nascer no Brasil* (Birth in Brazil) study] based on a sample of 23,894 interviewed puerperal women, clinical criteria were the most prevalent, followed by management and laboratorial criteria [16], which was in line with our results.

The main clinical criteria of MNMs observed were a respiratory rate higher than 40 bpm, shock and acute cyanosis. The main clinical criteria observed in the *Nascer no Brasil* study were a respiratory rate higher than 40 bpm, coagulation disorders and acute cyanosis. In another Brazilian study conducted at an intensive care unit (ICU), the most frequent clinical criteria were shock, a respiratory rate higher than 40 bpm and loss of consciousness for 12 or more hours [17]. These criteria are difficult to compare, particularly between different regions, because they are affected by clinical conditions that in turn are affected by socioeconomic conditions. However, in contrast to laboratorial and management criteria, clinical criteria are an extremely important tool for low-income regions, because no highly complex laboratory and hospital infrastructures are required.

The limitation to the use of laboratorial and management criteria is that most of these criteria require high complexity units for their use, and in some cases, near miss may be missed. This has been evaluated in a low resource settings and comparing low and high resource settings. Tailoring the WHO near miss criteria, such as lowering of number of packed red blood cell units or the including of disease-based criteria in low-resource settings were the necessary technology to classify patients as having near miss according to laboratorial or management criteria is not available seems to be a way to increase the accuracy of the near miss tool [18, 19].

Although ICU admission was not included in the WHO MNM criteria, it was an important marker of maternal severity in our study, because this criterion was identified in 94.5% of the pregnant women. ICU admission is included in the Mantel diagnostic MNM criteria [20]. However, due to the low availability of ICU beds and the non-uniform admission criteria in Brazil, ICU admission becomes a questionable marker because it is affected by the complexity of the services [8].

The distribution of MNMs by clinical condition showed that hypertension in pregnancy was the most frequent event (67.2%), corroborating the findings of other studies conducted in Brazil and in developing countries [10, 12, 15, 17]. Hypertension in pregnancy is also the main cause of direct obstetric mortality in Brazil and in several countries [21, 22]. Conversely, hemorrhage is the most frequent MNM event in developed countries [17].

Eclampsia was the most frequent hypertensive disorder (30.9%), with an MNM ratio of 16.9/1000 Lbs. However, severe preeclampsia and hemolysis, elevated liver enzymes, low platelet count (HELLP) syndrome has been reported in some studies as the most frequent event among hypertensive disorders [15, 17, 23]. Because our study was conducted at a high-risk maternity hospital, the most severe cases of hypertensive disorders, particularly eclampsia, might have been triaged to the unit. Furthermore, the high frequency of eclampsia might reflect the lack of quality in severe pre-eclampsia management with inadequate use of magnesium sulfate, because only 51.7% of these pregnant women used this medication before hospitalization. A randomized study conducted in 33 countries found that women who used magnesium sulfate had a 58% lower risk of eclampsia [24]. Thus, our study shows the need for professional training in early identification and adequate care for pregnant women with obstetric complications classified by the WHO as PLTCs to improve maternal morbidity and mortality rates.

We found no significant association between the sociodemographic profiles of mothers with and without adverse maternal outcomes in both the bivariate and the multivariate analyses. These results were expected considering the social strata of the pregnant women treated in the maternity hospital, who had virtually the same sociodemographic profile. However, a secondary analysis of a national cross-sectional study conducted in the Amazon and northeastern regions of Brazil analyzing the association between ethnic differences and the occurrence of MNMs showed that the occurrence of MNMs was higher among indigenous (53.1%) and black (28.4%) women than among white women [25].

Two sociodemographic profile issues were notable: 78.2% of the pregnant women with near miss events were non-white, and 83.7% had a family income of one minimum wage or lower. This finding shows that the variable “ethnicity” should be used as a social construct that is more closely related to environmental than genetic factors in medical and epidemiological studies, because genetic determination explains only a very small part of population disease and mortality [26]. In Brazil, racial inequality is clearly shown by differences in income. For example, blacks account for 90% of the poorest percentiles, whereas the percentage of Afro-descendants in the richest percentile is lower than 10% [27]. Maceió, which has a municipal human development index (MHDI) of 0.72, ranks last among the Brazilian state capitals, which highlights the relationship between poor socioeconomic conditions and a high incidence of adverse health events [28]. This result shows that fighting for improved quality of life is an efficient tool to decrease maternal morbidity and mortality in developing countries.

In our study, 18.2% of the pregnant women with MNMs had no prenatal visits, and only 34.6% had adequately made six or more prenatal visits. Making fewer than six prenatal visits significantly increased the risk of MNMs in the multivariate analysis by two-fold. Although prenatal care coverage and the number of medical visits per pregnant woman have increased over the last 15 years in Brazil [29], we still show quantitative and qualitative difficulties in prenatal care, which is a risk factor for MNMs [11, 17].

The incidence of cesarean sections continues to increase worldwide and reached 55% in Brazil in 2014 [30, 31]. Cesarean section in the current pregnancy had a high incidence in the group of pregnant women with MNMs (80%) and was significantly associated in the multivariate analysis, in which it increased the risk of occurrence of MNMs five-fold. This finding corroborates the results of other studies [10, 11, 17]. However, this association may be affected by confounding factors. In low-risk pregnancies, cesarean sections are known to pose potential risks to women’s health and may be a modifiable risk factor for maternal mortality when compared with vaginal delivery due to the increase in thromboembolism, puerperal infection, hemorrhages and anesthetic complications [30]. Conversely, in the case of MNMs, the high rates of cesarean sections may be found. Because this is a population of high-risk pregnant women who urgently require pregnancy resolution and cesarean section may more frequent.

A cesarean section is a surgical procedure that involves blood loss, anesthetic risks, and that raises risk of postoperative complications. Therefore, it seems thata cesarean section is an additional risk factor that increases riskof complications. Studies are controversial about how cesarean in the present pregnancy contribute to near miss cases [32, 33]. We believe that new studies should be performed to clarify the benefits and risks of the ideal level of cesarean sections to protect the mother and fetus in high-risk pregnancies.

Another indicator we used to assess the quality of maternal care was the “three delays model”. This model aims to identify when maternal care fails. Recent studies indicate that this model also explains a significant proportion of MNM cases [13, 34, 35]. Thus, the MNM approach and the structure of the three delays model may enhance health system monitoring and bridge gaps in emergency obstetric care [35].

In our study, although 69.1% of the pregnant women with MNMs showed some delay in care, the presence of this variable was not significantly associated with the outcome after the multivariate analysis. These results, which were in disagreement with recent studies, could be explained not only by the fact that the comparison groups in our study had the same sociodemographic conditions and were subject to the same risks, which could explain the first and second delays (which are associated with the characteristics of the women and their families), but also by the homogeneous distribution of the population treated at the maternity hospital. Conversely, the relatively low frequency of the third delay compared with other studies and its lack of association with the outcome in the present sample are surprising. The collection of data from medical records may have prevented accurate identification of delays in care, because the quality and quantity of information, especially in medical records of patients with care failures, are often insufficient for this analysis, and the interviews with the women may have not been elucidative [19, 36].

The strength of the study is that it is the first study on MNMs performed in the state of Alagoas, northeast Brazil, which is a region with high maternal morbidity and mortality rates. Additionally, the use of a multiple regression analysis model that considers interrelationships between variables decreases the occurrence of erroneous findings that may appear in the bivariate analysis. Moreover, we used WHO concepts and a one-year data collection period, which enabled us to capture seasonal variations.

Some limitations of this study were assessment of MNMs in a high-risk maternity hospital, which did not represent the base population of pregnant women in the state of Alagoas, and the collection of data on socioeconomic variables and access to care in interviews with patients, which might be subject to memory bias.

## Conclusions

In our study, the factors that remained associated with the occurrence of MNMs were fewer than six prenatal visits and cesarean section in the current pregnancy. These results highlight the need for improved quality and quantity of prenatal visits and the identification of innovative and viable models of labor and delivery care that value normal delivery and decrease the percentage of unnecessary cesarean sections.

## Data Availability

The data and material are with the corresponding author (Telmo Henrique Barbosa de Lima - E-mail: thbl@uol.com.br).

## Abbreviations

CIs: confidence intervals
CLAP: Latin American Centre of Perinatology
HDI: Human Development Index
ICU: Intensive Care Unit
LB: Live Births
MDGs: Millennium Development Goals
MDR: maternal death ratio
MESM: Santa Monica Maternity School Hospital
MNM: Maternal Near Miss
MNMR: maternal near miss ratio
NNM: Neonatal Near Miss
OR: Odds Ratio
PLTCs: Potentiallylife-threateningconditions
RR: Risk Ratio
SMOR: severe maternal outcome ratio
SUS: Unified Heath System
UN: United Nations
UNCISAL: State University of Health Sciences of Alagoas
WAMOs: with adverse maternal outcomes
WHO: World Health Organization
WOAMOs: women withoutadverse maternal outcomes
